# Preventive Inoculation

**Published:** 1899-07-15

**Authors:** 


### The Hospital

A Journal of

The Medical Sciences and Hospital Administration.

Vol. XXVI.—No. 668.] Edited by Sir Henry Burdett, K.C.B., and Solomon C. Smith, M.D., M.R.C.P. [July 15, 1899.

### Preventive Inoculation.

The steady spread and the wide acceptance of the practice of using the agents of disease and the life-products of disease germs in the treatment and for the prevention of fevers and other infective disorders, while it raises great hopes of our being able sooner or later to obtain a much more complete control over these diseases than we have at present, must on the other hand lead the careful inquirer to question greatly how far we are at present on the right track in regard to prophylaxis.

In the midst of great epidemics, whether of small-pox, cholera, or plague, one is driven to accept with thankfulness any help from any quarter which promises to cut short the prevalence of the pestilence or to diminish its fatality; but if one regards the course of events from the point of view of the spectator rather than the actor, and if one considers fevers and epidemics in their relation to the race rather than to the individual, one is driven to doubt greatly whether in the universal search for artificial prophylactics, which has of late taken possession of the medical world, medical science is following a true scent. That all knowledge is good we admit, and that all the facts which are now being accumulated in regard to the influence of the inoculation of man with disease germs and their products are likely to be of service in elucidating the truth in regard to immunity, resistance, and other phenomena, may well be granted, but we do not so readily grant, nor indeed do we believe for a moment, that real progress in public health will be found to lie in the direction of producing artificial immunity to disease by means of inoculation.

It is in accordance with all that we know as to the manner in which the stability of a species is maintained, to say that a race or a nation which exists under certain conditions depends for its continuance on the continuance of these conditions, and, although to the present generation, perhaps, it does not matter, those who take wide views can hardly be expected to shut their eyes to the almost necessary deduction that the inoculation syringe, if necessary now, must be a continuing condition, which is a somewhat serious prospect. Nor must we entirely forget that the survival of a race depends more upon fitness than upon numbers, and that the saving of the lives of vast numbers by aid of the syringe is by no means necessarily a step in the direction of permanence of race We are led to make these remarks not by any spirit of hostility to the researches which are at present going on, but because we think that at this moment, when, as is the case, hopes are being seriously held out of our being able to offer a prophylactic for a disease like typhoid fever, a natural product of our own sanitary sins, we stand at the parting of the ways, one of which leads to health by the narrow and difficult path of sanitary righteousness, and the other by the easy road of inoculation which—to express the tendency in extreme terms—would allow us to wallow in our filth and take no harm. For the last forty years, and up to the present time, a constant war has been made against filth of every kind, and especially against those kinds of filth which have been recognised to be a main cause of the prevalence of typhoid fever. And this war against filth has been of infinite service not only, perhaps not principally, in lessening the prevalence of that disease, but also in diminishing the mortality from a large number of other maladies. On every side we see proofs of the great advantage which a community derives from attacking that prime cause of so many diseases, the accumulation of excrementitious matter in its midst, whether in the form of foul air, foul water, or foul soil, and we are convinced that it is by continuing on this line, rather than in seeking for prophylactics, that safety is to be found.

Our main reason, indeed, for drawing attention to the present drift of tendencies is that at this moment the three diseases for which prophylaxis by means of inoculation is especially being sought are the three main filth diseases of the world—typhoid, cholera, and plague—diseases for the prevention of which we already possess other and, as we think, better ways. Small-pox stands on a different platform, and the success of vaccination in controlling a disease which has shown itself to be incapable of control in any other way cannot be taken as any argument in favour of preventive inoculations having for their aim the control of such diseases as typhoid fever, cholera, and plague, even if their efficacy for the purpose were better proven than it is. When we see groups of people, by virtue of their cleanly mode of life, able to walk practically unscathed through epidemics of plague; when we see cholera, which decimates certain towns, pass away harmless in the presence of a pure water supply; when we see typhoid fever being steadily uprooted from its strongholds in proportion as man takes care to keep clean the soil around his house and the water which he drinks; and, further, when we see that by aid of this cult of cleanliness even other diseases, which are by no means to be regarded as infectious, are lessened in their virulence; then can we understand how far from the true scent are those who would protect mankind from individual diseases by a series of separate preventive inoculations. As a means of protecting an army which must march into infected districts, and of immunising, if only for a time, those doctors, nurses, and hospital attendants who have to expose themselves to great risks, such measures may be justifiable, but as a means of protecting a people against the consequences of its own remissness—No!

